# Structure and Function Analyses of the Thioredoxin 2 and Thioredoxin Reductase Gene in Pacific White Shrimp (*Litopenaeus vannamei*)

**DOI:** 10.3390/ani15050629

**Published:** 2025-02-21

**Authors:** Tong Xu, Pei-Hua Zheng, Ke-Er Luan, Xiu-Xia Zhang, Jun-Tao Li, Ze-Long Zhang, Wei-Yan Hou, Li-Min Zhang, Yao-Peng Lu, Jian-An Xian

**Affiliations:** 1 Hainan Provincial Key Laboratory for Functional Components Research and Utilization of Marine Bio-Resources, Institute of Tropical Biosciences and Biotechnology, Chinese Academy of Tropical Agricultural Sciences, Haikou 571101, China; 18645437829@163.com (T.X.); zhengpeihua@itbb.org.cn (P.-H.Z.); claire9905@163.com (K.-E.L.); zhangxiuxia@itbb.org.cn (X.-X.Z.); lijuntao@itbb.org.cn (J.-T.L.); zhangzelong@itbb.org.cn (Z.-L.Z.); 18831687047@163.com (W.-Y.H.); 2 College of Biology and Agriculture, Jiamusi University, Jiamusi 154007, China; zhanglimin425@126.com

**Keywords:** thioredoxin system, gene expression, stress, RNA interference, *Litopenaeus vannamei*

## Abstract

The thioredoxin (Trx) system is one of the most significant systems in living organisms as it regulates cellular redox reactions and plays a pivotal protective role within the cell by promoting redox homeostasis. *Trx* and thioredoxin reductase (*TrxR*) are the core oxidoreductases of the Trx system. In this study, the novel full-length cDNAs of *LvTrx2* and *LvTrxR* were cloned from *Litopenaeus vannamei*. The ORFs of *LvTrx2* and *LvTrxR* were 453 bp and 1785 bp, encoding polypeptides consisting of 150 and 596 amino acids. Sequence alignment analysis revealed that the amino acid sequence of *LvTrx2* shared a high degree of identity (93%) with that of *Penaeus chinensis*, while in *LvTrxR*, it exhibited a similarity level of 95% with previously submitted *Penaeus chinensis* and *Penaeus monodon* sequences. Regarding tissue-specific expression patterns, *LvTrx2* showed its highest expression levels in hepatopancreas and gill. For *LvTrxR*, the highest expression was observed in gill followed by hepatopancreas and intestine. During exposure to ammonia-N, there was a significant upregulation in the relative mRNA levels of *LvTrx2* and *LvTrxR* in hepatopancreas and gill, with the peak values occurring at 24 h or 48 h of exposure. After LPS injection, the *LvTrx2* and *LvTrxR* transcripts in hepatopancreas and gill had different upregulated levels. These findings suggest that *LvTrx2* and *LvTrxR* play pivotal roles in enhancing stress resistance and bolstering antibacterial defense mechanisms in *L. vannamei*. To explore the roles, *LvTrx2* expression was knocked down in vivo to verify the defense mechanism against 4-NP stress. *LvTrx2* silencing in 4-NP-challenged shrimp could significantly induce the gene expression of antioxidant-related genes (except for *LvTrxR*) and aggravate the oxidative damage of lipids. This study suggests that the Trx system is involved in regulating the antioxidant processes, and *LvTrx2* and *LvTrxR* play a vital role in defense responses against environmental stress.

## Simple Summary

In this study, the full-length open reading frames (ORFs) of thioredoxin 2 (*Trx2*) and thioredoxin reductase (*TrxR*) were successfully cloned from the Pacific white shrimp (*Litopenaeus vannamei*). They were highly expressed in the hepatopancreas and gill. Through ammonia-N stress or lipopolysaccharide (LPS) injection, we found out that *LvTrx2* was involved in the immune defense response process of stress resistance and antibacterial activity, *LvTrxR* was involved in antioxidant defense processes, and 4-nonylphenol (4-NP) stress after silencing *LvTrx2* caused an increase in the oxidative damage level in lipids. Glutaredoxin 2 (*Grx2*), *Grx3*, glutathione peroxidase (*GPx*), and glutathione S-transferase (*GST*) were upregulated and may have a synergistic effect with *LvTrx2*. These results indicate that the Trx system participates in regulating antioxidant processes, and *LvTrx2* and *LvTrxR* play a crucial role in defending environmental stress.

## 1. Introduction

Pacific white shrimp (*Litopenaeus vannamei*) is known as one of the three highest-production shrimp in the world, which is the most economic aquaculture species [1]. In China, the yields of *L. vannamei* occupied the first position in shrimp mariculture [2]. Due to the development of industrialization and intensive culture systems, the degradation of aquaculture environments has become a significant factor affecting *L. vannamei* over the past two decades. The *L. vannamei* species residing in benthic zones of ponds are susceptible to the influence of environmental pollutants (ammonia nitrogen [3], organic pesticides [4], heavy metal [5], etc.) and various other factors (temperature [6], salinity [7], pH [8], etc.). Thus, it is imperative to comprehend the resistance mechanisms of *L. vannamei* against environmental pollutants.

The thioredoxin (Trx) system is a nicotinamide adenine dinucleotide phosphate (NADPH)-dependent reductase system, consisting of *Trx*, thioredoxin reductase (*TrxR*), NADPH, and thioredoxin interacting protein (*Txnip*). The Trx system regulates cellular redox reactions and plays a pivotal protective role within the cell by promoting redox homeostasis [9,10]. Trx and TrxR are the core oxidoreductases of the Trx system. Trx is a small disulfide reductase (approximately 12 kDa) that belongs to the thioredoxin superfamily. Trx plays an important role in maintaining the delicate balance of oxidative stress and safeguarding cells against oxidative damage [11]. Trx consists of two distinct types: cytosolic Trx (Trx1) and mitochondrial Trx (Trx2) [12]. Trx1 includes three additional Cys residues apart from the two in the active center, which is the predominant form of Trx involved in physiological processes (e.g., cell growth, apoptosis, and inflammatory response). Trx2 possesses two Cys residues at its active site. Its precursor protein carries a mitochondrial localization sequence, and mature Trx2 is localized within the mitochondria [13,14]. The conserved active site of most Trx is Cys-Gly-Pro-Cys, which can catalyze the progress of many redox reactions. Their primary function is to participate in a series of physiological and biochemical reactions, such as redox reactions, by reducing disulfide bonds on intracellular target proteins [15,16]. TrxR, weighing between 55 and 60 kDa, is a selenium-containing dimeric flavoprotein with an NADPH domain and has broad substrate specificity [17]. TrxRs are the sole known enzymes capable of reducing Trxs, establishing TrxR as an essential component of Trx functionality [18]. The relatively conservative domain of TrxR is Cys-Val-Asn-Val-GLy-Cys, close to the FAD domain. In vivo, TrxR eliminates reactive oxygen species (ROS) while maintaining cellular and tissue homeostasis internally and peripherally. Mammalian cells contain three distinct types of this enzyme: TrxR1, TrxR2, and thioredoxin glutathione reductase (TGR) [11].

In recent years, *Trx* and *TrxR* have garnered significant attention due to their diverse and potent functions. They have a crucial involvement in oxidative stress regulation, cell growth, proliferation, apoptosis, and signal transduction pathways [11]. In *Phascolosoma esculenta*, the recombinant Trx2 protein exhibited antioxidant activity and enhanced the cadmium (Cd) tolerance of *Escherichia coli*. Following *Trx2* interference, alterations in the expression of apoptosis-related genes were observed, proving that PeTrx2 played an important role in antioxidant and anti-apoptosis *P. esculenta* [19]. Previous research on *Hippocampus abdominalis* has demonstrated that *HaTrx-2* exhibits significant antioxidant and free radical scavenging properties, as evidenced by cell viability assays, DPPH radical scavenging activities, and MCO assays [20]. For *Sinopotamon henanense*, *TrxR* participates in the redox process under Cd exposure [21]. A limited number of investigations have focused on decapods. This current study presents the initial cloning of full-length sequences for *Trx2* and *TrxR* from *L. vannamei* with subsequent molecular characterization analysis. To investigate the biological functions of *Trx2* and *TrxR*, the expression patterns of *Trx2* and *TrxR* were examined in Pacific white shrimp under ammonia-N stress and following lipopolysaccharide (LPS) injection. Additionally, *Trx2* was silenced to elucidate its role in *L. vannamei* after exposure to 4-nonylphenol (4-NP) stress.

## 2. Materials and Methods

### 2.1. Experimental Shrimp

Healthy Pacific white shrimp *L. vannamei* were purchased from a local shrimp farm in Haikou, China, with an average body weight of 8.22 ± 1.18 g and reared in cycling filtered plastic tanks in salinity 20‰, temperature 22–23 °C, and pH 7.9–8.0 conditions. Before the stress experiment started for 12 h, the shrimp were fed a commercial shrimp diet (40.0% protein, 5.0% fat, 5.0% fiber, and 16.0% ash) twice daily.

### 2.2. Ammonia-N Stress and LPS Challenge

Expression changes in *LvTrx2* and *LvTrxR* in shrimp were determined under ammonia-N stress or LPS injection. Two ammonia-N doses (zero and 20 mg L^−1^) were administered in each ammonia-N exposure experiment. The 20 mg L^−1^ ammonia-N solution was prepared using ammonium chloride (NH_4_Cl) (Guangzhou Chemical Reagent Factory, Guangzhou, China) to 20‰ seawater [22]. The actual mean doses of ammonia-N for the control and test groups were 0.01 and 20.32 mg L^−1^, respectively. Three replicates were set for the test and control groups under ammonia-N stress, respectively. Each replicate had 25 shrimp in plastic tanks with 80 L of water (22–23 °C, pH 7.9–8.0, salinity 20‰) aerated continuously using air stones. The seawater was renewed daily.

The LPS (2 mg mL^−1^) from *Escherichia coli* (055: B5, Sigma, Burlington, MA, USA) was dissolved in physiological saline solution (0.85% NaCl) to achieve a dosage of 2 μg μL^−1^. The *L. vannamei* were allocated into two groups, with 25 shrimp per replicate. The LPS injection dose of the test groups was 8 μg g^−1^ wet weight based on a previous study [23]. The control shrimp received an equivalent amount of sterile physiological saline solution.

After 0, 3, 6, 12, 24, and 48 h of ammonia-N stress or LPS injection, nine shrimp were collected from each group. Before RNA extraction, the hepatopancreas and gill of shrimp were rapidly dissected and preserved in liquid nitrogen followed by storage at −80 °C.

### 2.3. RNA Extraction and Complementary DNA (cDNA) Synthesis

The tissue samples were pulverized in liquid nitrogen followed by the extraction of total RNA using TRIzol reagent (Invitrogen, Waltham, MA, USA) as per the manufacturer’s protocol. The extracted RNAs were treated with RNase-free DNase I (TakaRa, Kusatsu, Shiga, Japan). The quantity and quality of each RNA were measured using a NanoDrop 2000 spectrophotometer (NanoDrop Technologies, Wilmington, DE, USA). Agarose electrophoresis (1%) was performed to verify the integrity of the RNA. First-strand cDNA was synthesized from total RNA using the PrimeScript RT reagent kit with a gDNA Eraser (Takara, Dalian, China).

### 2.4. Cloning of Trx2 and TrxR and Sequence Analysis

A small fragment of *Trx2* and *TrxR* cDNA was derived from the transcriptome data we previously acquired for *L. vannamei*. The Basic Local Alignment Search Tool (BLAST, https://blast.ncbi.nlm.nih.gov/Blast.cgi, accessed on 10 January 2024) analysis of all the expressed sequence tags (ESTs) from a cDNA library of multiple species revealed a high degree of similarity between the EST and the previously identified *Trx2* and *TrxR*. The first-round polymerase chain reaction (PCR) was performed using *Trx2* primers (*Trx2* F1 and R1) or *TrxR* primers (*TrxR* F1 and R1) (Table 1). The PCR conditions included 1 cycle of denaturation at 94 °C for 3 min, followed by 35 cycles of amplification consisting of denaturation at 94 °C for 30 s, annealing at 58 °C for 30 s, and extension at 72 °C for 1 min. A final extension step was carried out at 72 °C for 10 min.

The PCR products were cloned into the pMD18-T Vector (Takara, Dalian, China) and subjected to sequencing analysis conducted by the Beijing Genomics Institute (BGI) (Guangzhou, China).

### 2.5. Rapid Amplification of cDNA Ends (RACE)

Based on the obtained small sequence of *L.vannamei Trx2* and *TrxR*, specific primers for the 3′ and 5′ ends were designed using the 3′-Full RACE Core Set with PrimeScript RTase (Takara, Dalian, China) and the SMART RACE cDNA Amplification Kit (Clontech, Mountain View, CA, USA). The primer sequences are provided in Table 1.

The PCR for the 3′ end was performed using gene-specific primer *Trx2*-3′F1 or *TrxR*-3′F1 and the 3′ RACE Outer Primer. The PCR was in a 50 µL reaction volume containing 2 µL of 3′-RACE-Ready cDNA, 8 µL of 1 × cDNA Dilution Buffer II, 2 µL of 3′ RACE Outer Primer (10 µM), 2 µL of Gene-Specific Outer Primer (10 μM), 4 µL of 10 × LA PCR Buffer II (Mg^2+^ Free), 3 µL of MgCl_2_ (25 mM), 0.25 µL of TaKaRa LA Taq (5 U/µL), and 28.75 µL of dH_2_O. The PCR protocol consisted of an initial denaturation step at 94 °C for 3 min, followed by 30 cycles of denaturation at 94 °C for 30 s, annealing at 58 °C for 30 s, extension at 72 °C for 1 min, and a final extension step at 72 °C for 10 min.

The 5′-untranslated regions (UTRs) were amplified using *Trx2*-5′R1 or *TrxR*-5′R1 and universal primer mix (UPM). The PCRs include 2.5 µL of 5′-RACE-Ready cDNA, 5 µL of 10 × UPM, 1 µL of 5′ Gene-specific primer *TrxR*-5′R1 or *TrxR*-5′R1, 15.5 µL of PCR-Grade H_2_O, 25 µL of 2× SeqAmp Buffer, and 1 µL of SeqAmp DNA Polymerase. The PCR was performed at 94 °C for 30 s, 68 °C for 30 s, and 72 °C for 3 min. We 30 PCR cycles first as described and analyzed 5 µL from each tube, along with the appropriate DNA size markers, on a 1.2% agarose gel. Electrophoresis was performed for 10 min at 20 V/200 mA, after which SYBR^®^ Green I (Molecular Probes, Eugene, OR, USA) staining was used to visualize the PCR products. The gel-purified products of the 5′- and 3′-RACE PCR were cloned and sequenced according to the described protocol. All primer sequences can be found in Table 1.

### 2.6. Bioinformatics Analysis

The open reading frame (ORF) and amino acid sequences were analyzed using the software EditSeq (v7.1). The nucleotide and amino acid sequences of *Trx2* and *TrxR* cDNA were analyzed via the BLAST algorithm at The National Center for Biotechnology Information (NCBI) website (https://blast.ncbi.nlm.nih.gov/Blast.cgi, accessed on 8 March 2024) and Expasy search program (http://au.expasy.org/tools/, accessed on 8 March 2024). Multiple sequence alignment was performed using Clustal X software (1.83). Subcellular localization of proteins was analyzed using TargetP. SignalP 4.1 software (https://services.healthtech.dtu.dk/services/SignalP-4.1/, accessed on 9 March 2024) was used to predict the signal peptide. NetPhos 3.1 software (https://services.healthtech.dtu.dk/services/NetPhos-3.1/, accessed on 9 March 2024) was used for the phosphorylation site analysis. Based on NCBI BLAST, a diverse set of sequences from invertebrates to vertebrates, exhibiting moderate-to-high sequence similarity (45–95%), were utilized to construct the phylogenetic tree. Neighbor-joining phylogenetic tree based on the deduced amino acid sequences of *Trx2* and *TrxR* was constructed using Molecular Evolutionary Genetics Analysis (MEGA) 6.0 software. Bootstrap sampling was performed with 1000 replicates, and branches with less than 40% bootstrap support were collapsed. Evolutionary distances were calculated using the Poisson correction method.

### 2.7. Tissue Expression

We randomly selected nine shrimp to collect samples. For each shrimp, a 25-gauge needle and 1.5 mL syringe were used to collect hemolymph (400 μL) from the pericardial sinus, and the same volume of ice-cold anticoagulant solution (AS, glucose 20.5 g L^−1^, sodium citrate 8 g L^−1^, sodium chloride 4.2 g L^−1^, pH 7.5) was absorbed. Hemocytes were separated by centrifugation at 800× *g* and 4 °C for 5 min, and the hemocyte pellets were used for RNA extraction. The same shrimp were dissected out of the eyestalk, gill, hepatopancreas, muscle, and intestine and preserved in liquid nitrogen for RNA extraction.

Using the house-keeping gene *β-actin* as an internal control, the relative expression of *LvTrx2* or *LvTrxR* was determined in various tissues (gill, hepatopancreas, eyestalk, intestine, muscle, and hemocytes) through quantitative real-time PCR (qRT-PCR).

### 2.8. Gene Expression Analysis Under Different Stress Conditions

Following the previously described method for RNA extraction and cDNA synthesis, subsequent amplification was performed. After ammonia-N stress and LPS injection, a qRT-PCR using Stratagene Mx3005P (Agilent, Santa Clara, CA, USA) was employed to investigate the expression of *LvTrx2* and *LvTrxR* in the hepatopancreas and gill (Table 1). The results were quantified using the 2^−ΔΔCt^ method [24]. All data are presented as means ± standard deviation (SD).

### 2.9. LvTrx2 Gene Silence Experiment

#### 2.9.1. Double-Stranded RNA (dsRNA) Synthesis

The DNA templates for the dsRNA preparation were generated using gene-specific primers, *Trx2*i-F and *Trx2*i-R, containing a T7 promoter sequence at the 5′ end (Table 1). To serve as a control, green fluorescent protein (GFP) dsRNA was amplified from a pGFP vector template using primers with the T7 promoter sequences indicated in Table 1. The PCR products were purified using the QIAquick PCR Purification Kit (QIAGEN, Hilden, Germany) and utilized as templates for synthesizing *LvTrx2* or GFP dsRNA with the T7 RiboMAXTM Express RNAi System (Promega, Madison, WI, USA). The synthesized dsRNAs were validated through agarose electrophoresis and their concentrations were estimated by spectrophotometry at an absorbance of 260 nm.

#### 2.9.2. Gene Silencing and qRT-PCR

In the RNA interference (RNAi) experiment, dsRNAs were dissolved in a protective buffer (10 mM Tris-HCl, pH 7.5, 400 mM NaCl) to achieve a final dose of 2.5 μg μL^−1^. The shrimp were divided into two groups and injected with *LvTrx2* (2.5 µg g^−1^ shrimp) or *GFP* dsRNA (2.5 µg g^−1^ shrimp) at the lateral region of the fourth abdominal segment [25]. The hepatopancreas were individually collected from twenty shrimp at time points of 0, 24, 48, 72, and 96 h after dsRNA injection. The transcript level of the *Trx2* gene in each RNA sample was examined by qRT-PCR using specific primer pairs *Trx2*-RT-F1 and *Trx2*-RT-R1 (Table 1).

#### 2.9.3. Effects of 4-NP Stress on LvTrx2-Interfered Shrimp

To evaluate the impact of 4-NP stress on *L. vannamei* following *LvTrx2* suppression, shrimp were randomly allocated into two experimental groups. One group received an injection of *GFP* dsRNA as a control, while the other group was injected with *Trx2* dsRNA. Twenty-four hours after the injection, all groups of shrimp were exposed to 4-NP (500 µg L^−1^). Then, the samples were collected 24 h after the injection and 0, 1.5, 3, 6, 12, and 24 h after exposure. At each point, thirty shrimp from each group were randomly selected for hepatopancreas collection. The hepatopancreas was rapidly frozen in liquid nitrogen and stored at −80 °C before RNA extraction.

#### 2.9.4. Expression Levels of LvTrx2 and Antioxidant-Related Genes

Nine shrimp were collected from each time point, and the hepatopancreas of the shrimp was rapidly dissected and preserved in liquid nitrogen followed by storage at −80 °C. The relative expression levels of *LvTrx2*, *LvTrxR*, and several other antioxidant-related genes (*GPx*, *GST*, glutaredoxin 2 (*Grx2*), (*Grx3*)) were determined using qRT-PCR in *LvTrx2*-silenced shrimp and control shrimp (ds*GFP*).

#### 2.9.5. Malondialdehyde (MDA) Content

The MDA content was utilized to evaluate the degree of lipid peroxidation through the thiobarbituric acid (TBA) assay under conditions of oxidative stress. The MDA content in the hepatopancreas was measured using commercial kits (Nanjing Jiancheng Bioengineering Institute, Nanjing, China) according to the manufacturer’s instructions. The MDA content in the lipid hydroperoxide decomposition products could react with TBA to form red compounds exhibiting an absorption peak of 532 nm. The data underwent triplicate testing. The average of the three values was utilized for the data analysis.

### 2.10. Statistical Analyses

The data were reported as means ± SD. The normality of the data was assessed using the Shapiro–Wilk test. A one-way analysis of variance [26] was employed to analyze the data. A multiple comparison (Tukey) test was conducted to compare significant differences among the treatments using the Statistical Package for the Social Sciences (SPSS) 18.0 software (SPSS Inc., Chicago, IL, USA). A significance level of *p* < 0.05 was considered statistically significant.

## 3. Results

### 3.1. Cloning and Sequence Analysis of LvTrx2 and LvTrxR Genes

**Figure 1:**
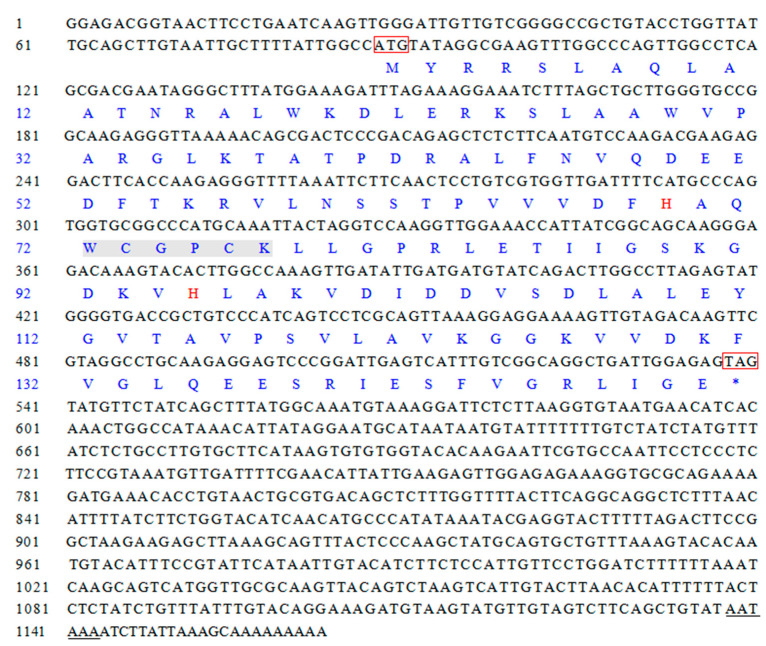
Nucleotide and amino acid sequences of *L. vannamei Trx2* gene. The letters in the box represent the initiation codon (ATG) and a termination codon (TAG), while the underlined region indicates the polyadenylation signal sequence (AATAAA). The conserved domain (W-C-G-P-C-K) is highlighted in gray, and two relatively non-conservative histidine residues (H) are displayed in red font. * indicates translation termination.

The cDNA template was derived from the hepatopancreas of *L. vannamei*. The full-length cDNA of *LvTrx2* was 1165 bp, comprising an 87 bp 5′-UTR and a 625 bp 3′-UTR, and it exhibited a typical polyadenylation signal AATAAA repeat and a poly(A) tail. The complete ORF of *LvTrx2* spanned 453 bp, corresponding to a coding sequence for 150 amino acids. The protein encoded by LvTrx2 exhibited a molecular weight of 16.46 kDa, a theoretical pI of 7.76, and an instability index of 29.79, classifying it as a stable protein. The LvTrx2 protein contained 41 charged amino acid residues, with 20 being negatively charged and 21 positively charged. It exhibited an aliphatic index of 99.40 and an average hydrophilicity value of −0.109. The protein was predicted to possess 10 phosphorylation sites, consisting of 7 serine and 3 threonine (Thr) residues. The absence of a signal peptide in the LvTrx2 protein sequence was observed. In terms of secondary structure prediction, α-helix accounted for 30.67% (46 amino acids) and extended strand accounted for 22.67% (34 amino acids), while random coil constituted the majority at 46.67% (70 amino acids) (Figure 1).

**Figure 2:**
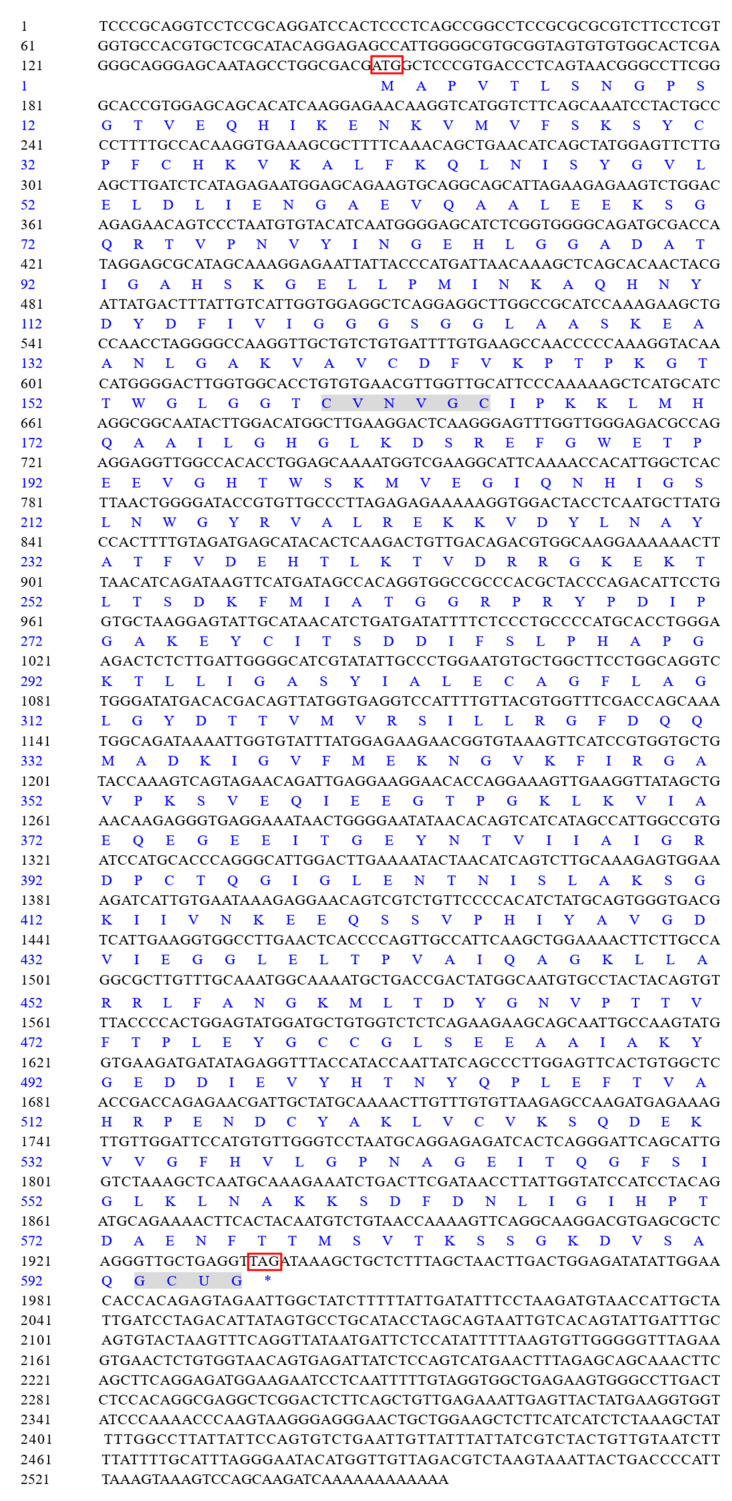
Nucleotide and amino acid sequences of *L. vannamei TrxR* gene. The letters in the box indicate the initiation codon (ATG) and the termination codon (TAG). The conserved domains C-V-N-V-G-C and G-C-U-G are highlighted in gray. * indicates translation termination.

The full-length cDNA of *LvTrxR* was 2554 bp, consisting of a 146 bp 5′-UTR and a 618 bp 3′-UTR. The ORF of *LvTrxR* was 1785 bp, encoding a polypeptide of 596 amino acids. The *LvTrxR*-encoded protein exhibited a molecular weight of 64.34 kDa, a theoretical pI of 5.87, and an instability index of 30.13, which classified it as a stable protein. The LvTrxR protein contained 71 negatively charged and 60 positively charged residues. It exhibited an aliphatic index of 85.89 and an average hydrophilicity value of −0.180. The protein was predicted to contain 23 Ser, 20 Thr, and 6 tyrosine (Tyr) phosphorylation sites. The LvTrxR protein lacks signal peptide. In terms of secondary structure prediction, the α-helix constituted 26.01% (155 amino acids), the extended strand accounted for 26.34% (157 amino acids), β-turn represented 12.58% (75 amino acids), while the random coil comprised the majority at 35.07% (209 amino acids). A high abundance of glycine residues was observed in the polypeptide, accounting for 11.20% (67 amino acids) (Figure 2).

### 3.2. Multiple Alignment and Phylogenetic Analysis

The blast analysis revealed that LvTrx2 exhibited the highest similarity with Trx2 from two species of shrimp, Chinese shrimp (*Penaeus chinensis*, XP_047475382.1) and giant tiger prawn (*Penaeus monodon*, XP_037785345.1), showing 93% identity in both cases. Additionally, LvTrx2 shared a significant homology with the Chinese mitten crab (*Eriocheir sinensis*) (XP_050712400.1), displaying 85% identity. Notable similarities were observed with the mud crab (*Scylla paramamosain*) (AFW97641.1) and gazami crab (*Portunus trituberculatus*) (XP_045121346.1), exhibiting 84% identity each, as well as oriental river prawn (*Macrobrachium nipponense*) (AQW44864.1) with 71% identity. The sequence information used for alignment and constructing evolutionary trees is supplemented in Table S1.

The BLAST analysis indicated that LvTrxR shared the highest similarity with TrxR from *P. chinensis* (XP_047469221.1, 95%), *P. monodon* (XP_037782185.1, 95%), and *Penaeus indicus* (XP_063611883.1, 95%). The second closest similarity was found with the karuma shrimp (*Penaeus japonicus*) (XP_042872148.1, 93% identity). This was followed by the American lobsters (*Homarus americanus*) (XP_042238035.1), crayfish (*Procambarus clarkii*) (XP_045595557.1), and swimming crab (*P. trituberculatus*) (XP_045134908.1), with similarities of 81%, 78%, and 77%, respectively. Additional sequence information used for alignment and constructing evolutionary trees is found in Table S1.

**Figure 3:**
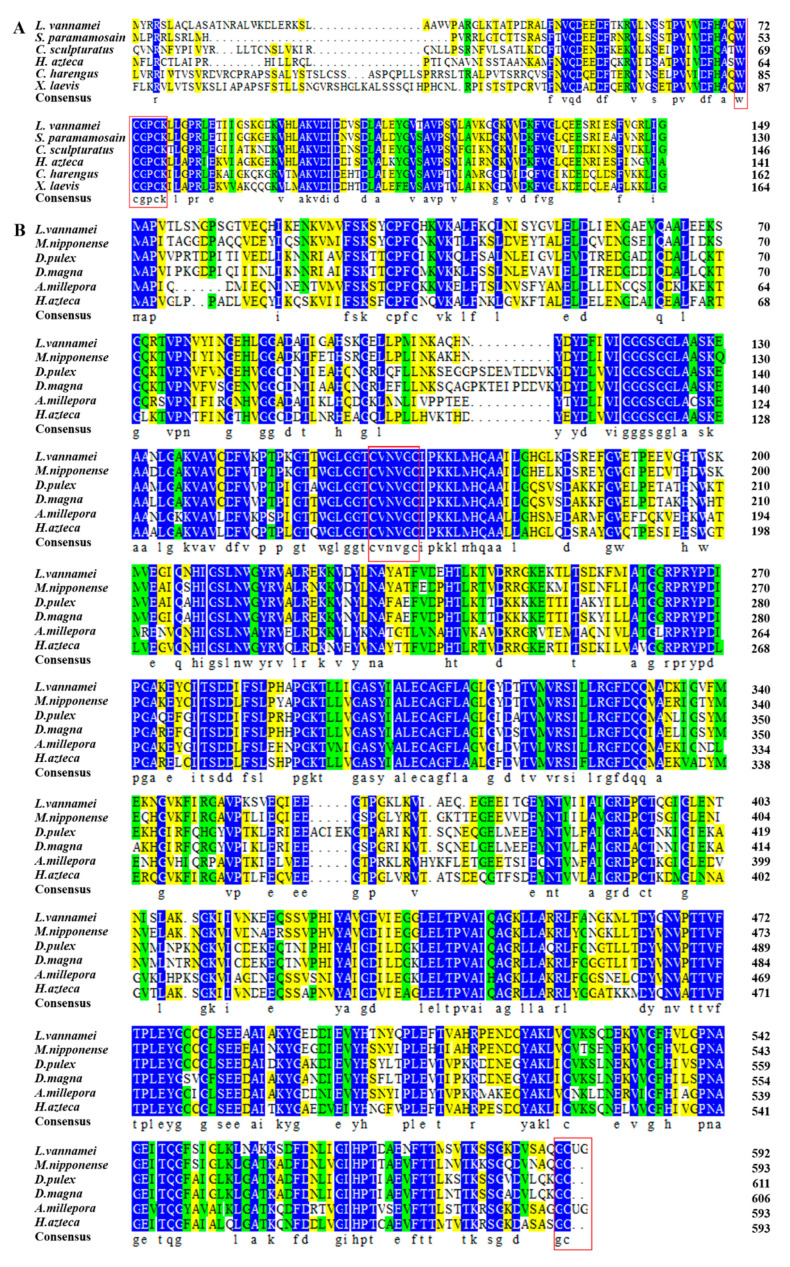
Multiple sequence alignments of LvTrx2 (**A**) and LvTrxR (**B**). The blue region indicates that all sequences share the same amino acid residue. The square shows the conserved domain of LvTrx2 and LvTrxR. The blue region indicates all sequences shared the same amino acid residue, the green region indicates all sequences shared ≥75% of the same amino acid residue, and the yellow region indicates all sequences shared ≥50% of the same amino acid residue.

Multiple sequence alignment has revealed that LvTrx2 contains a conserved domain specific to the Trx family, which is critical for the fundamental structure and function of the Trx protein. The Trp-Cys-Gly-Pro-Cys-Lys (W-C-G-P-C-K) structural domain, the Trx family activate area, is highly conservative in Trx2 for all of the compared species. Using TargetP, the subcellular localization of proteins was analyzed, and LvTrx2 amino acid sequences had mitochondrial targeting peptide (mTP), which indicates that it plays a role in mitochondria (Figure 3A).

Through multiple sequence alignment, LvTrxR contains a conserved domain Cys-Val-Asn-Val-Gly-Cys (C-V-N-V-G-C), which is specific to the TrxR protein family. The conserved domain plays a significant role in fundamental architecture and exhibits high conservation across all of the species that were compared. The sequence terminates with an unusual amino acid called selenocysteine (Sec, U), which is present in Gly-Cys-Sec-Gly (G-C-U-G) (Figure 3B).

**Figure 4:**
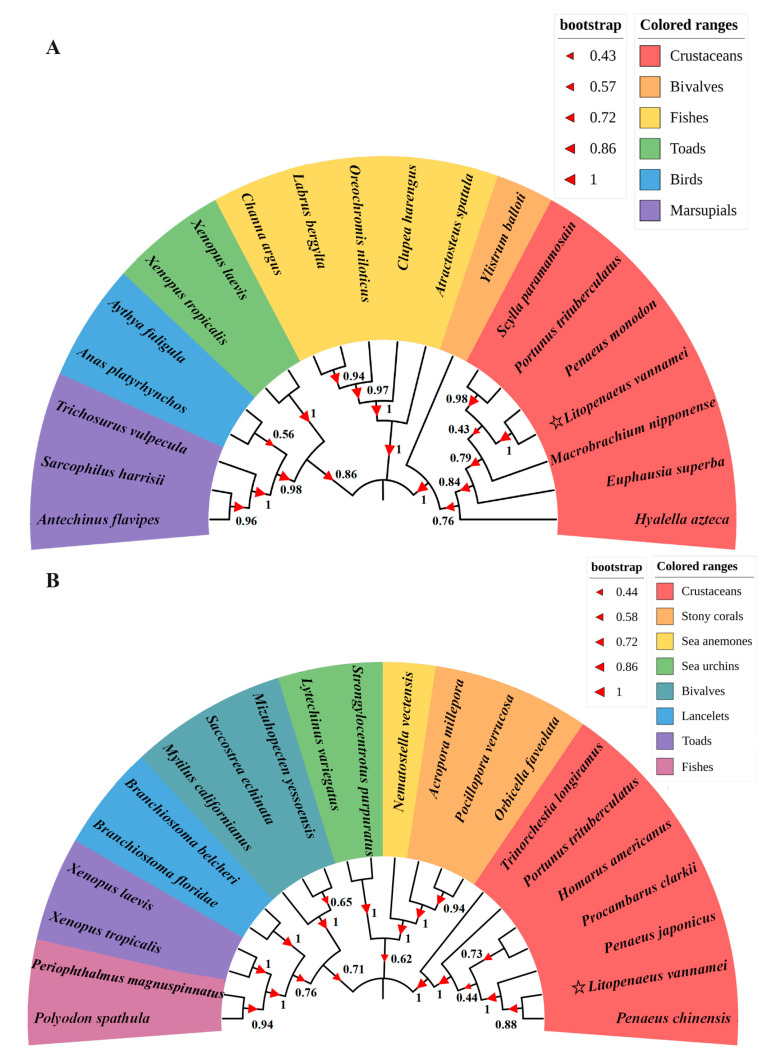
Phylogenetic analysis of *L. vannamei* LvTrx2 (**A**) and LvTrxR (**B**).

Phylogenetic tree analysis was conducted to investigate the evolutionary relationship between LvTrx2 or LvTrxR and the chosen vertebrates and invertebrates, providing insights into their inter-relationships. The phylogenetic tree exhibited congruence with the taxonomic classification of the species. *L. vannamei* clustered with *P. monodon* and *P. chinensis*, which belong to crustaceans (Figure 4).

### 3.3. Tissue Expression in L. vannamei

**Figure 5:**
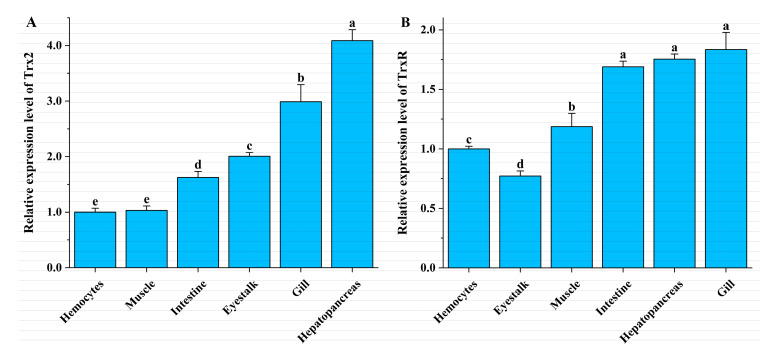
Expression levels of *LvTrx2* (**A**) and *LvTrxR* (**B**) genes in different tissues of *L. vannamei*. Data in the same group with different letters are significantly different (*p* < 0.05).

qRT-PCR showed the ubiquitous presence of *LvTrx2* and *LvTrxR* in all examined tissues of *L. vannamei*, including gill, hepatopancreas, eyestalk, intestine, muscle, and hemocytes. *LvTrx2* exhibited expression levels in the hepatopancreas, gill, and eyestalk, followed by the intestine, whereas its expression level was lowest in muscle and hemocytes (Figure 5A). *LvTrxR* displayed the highest expression levels in gill, hepatopancreas, and intestine, and moderate levels were observed in muscle and hemocytes, while its expression level was relatively low in the eyestalk (Figure 5B).

### 3.4. Expression Profiles of LvTrx2 and LvTrxR in Hepatopancreas and Gill During Ammonia-N Stress

**Figure 6:**
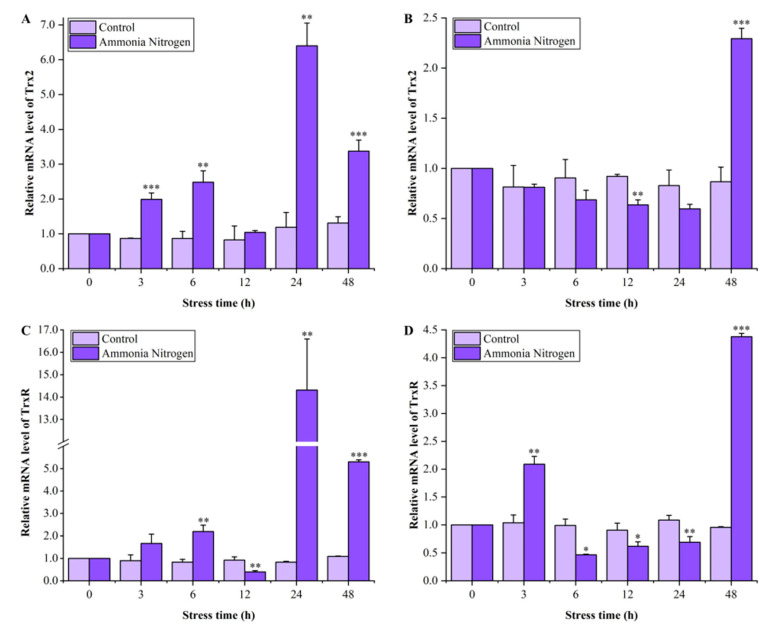
Expression levels of *LvTrx2* in the hepatopancreas (**A**) and gill (**B**) and *LvTrxR* in the hepatopancreas (**C**) and gill (**D**) of *L. vannamei* under ammonia-N stress. Statistical significance was calculated using SPSS 18.0 (* stands for *p* < 0.05, ** stands for *p* < 0.01, *** stands for *p* < 0.001).

During exposure to ammonia-N, the *LvTrx2* transcripts in the hepatopancreas exhibited significant upregulation after 3 and 6 h (*p* < 0.01). At 24 and 48 h, there was an extremely significant upregulation (*p* < 0.01), with the peak value observed after 24 h of exposure being approximately 5.4 times higher than that of the control group (Figure 6A). The *LvTrx2* transcripts in the gill exhibited no significant change at 3–6 h, which were significantly inhibited after 12 h of exposure (*p* < 0.01), and these transcripts returned to levels comparable to the control group at 24 h. The *LvTrx2* expression was significantly upregulated at 48 h (*p* < 0.001), which was about 2.65 times higher than the control group (Figure 6B).

The relative expression level of the *LvTrxR* gene in the hepatopancreas of *L. vannamei* under ammonia-N stress was slightly upregulated at 3–6 h (*p* < 0.01), followed by a significant downregulation at 12 h (*p* < 0.01). After 24 h and 48 h of stress, the expression level increased sharply (*p* < 0.01) and reached its peak at 24 h, which was 17.13 times that of the control group (Figure 6C). The relative expression of the *LvTrxR* gene in the gill tissue showed a similar trend to that of the hepatopancreas. At the initial stage (3 h), the expression level of *LvTrxR* increased slightly (*p* < 0.01) and then decreased significantly (6–24 h) (*p* < 0.05). At 48 h, the expression level increased sharply and was extremely significant (*p* < 0.001). Notably, the *LvTrxR* gene expression level in the gill peaked at 48 h, exhibiting a significant increase of 4.57-fold compared to that observed in the control group (Figure 6D).

### 3.5. Expression Profiles of LvTrx2 and LvTrxR in Hepatopancreas and Gill After LPS Injection

**Figure 7:**
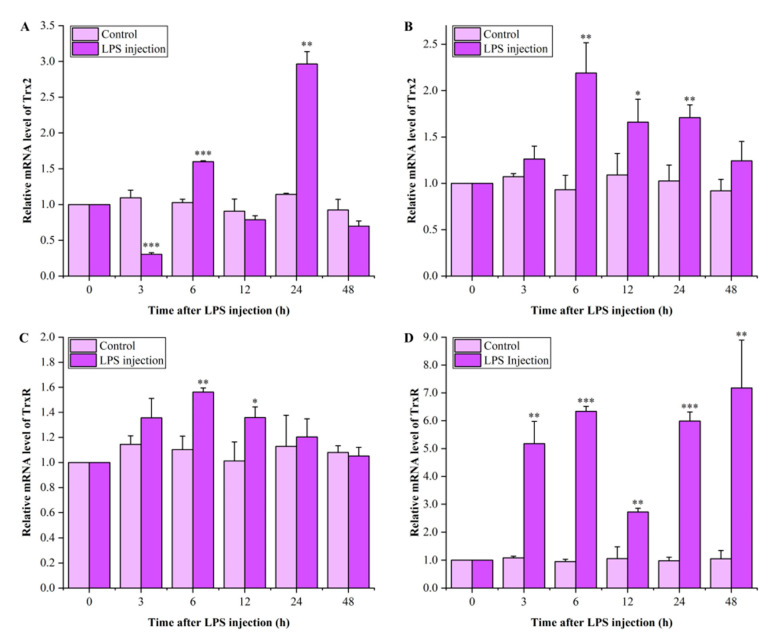
Expression levels of *LvTrx2* in the hepatopancreas (**A**) and gill (**B**) and *LvTrxR* in the hepatopancreas (**C**) and gill (**D**) of *L. vannamei* after LPS injection. Statistical significance was calculated using SPSS 18.0 (* stands for *p* < 0.05, ** stands for *p* < 0.01, *** stands for *p* < 0.001).

The transcriptional level of *LvTrx2* in the hepatopancreas extremely significantly decreased 3 h after the injection (*p* < 0.001). It rebounded after 6 h of stimulation, showing a significantly higher expression compared to the control group (*p* < 0.001). After 12 h of stress, the *LvTrx2* expression was restored to levels like the control group (*p* > 0.05). The expression level of *LvTrx2* was significantly increased at 24 h of stress (*p* < 0.001) and reached its peak value, which was about 2.60 times that of the control group. After 48 h of stress, the *LvTrx2* expression level returned to levels comparable with the control group (*p* > 0.05) (Figure 7A). The expression level of *LvTrx2* in the gill showed no change at 3 h after exposure (*p* > 0.05), followed by a significant upregulation of the expression levels after 6–24 h of exposure (*p* < 0.05). The peak expression level was observed at 6 h, approximately 2.35 times higher than the control group. By 48 h post-LPS injection, the expression levels had returned to those of the control group (*p* > 0.05) (Figure 7B).

The expression level of *LvTrxR* in the *L. vannamei* hepatopancreas showed no significant change after 3 h of LPS injection. At 6–12 h, the levels of *LvTrxR* were significantly higher than those in the control group (*p* < 0.05) and returned to similar levels as the control group (*p* > 0.05) (Figure 7C). After LPS injection, the expression level of *LvTrxR* in the gill significantly increased within 48 h after stimulation (*p* < 0.01), reaching a peak at 48 h with a 6.81-fold increase compared to the control group (Figure 7D).

### 3.6. LvTrx2 Silencing Assay

**Figure 8:**
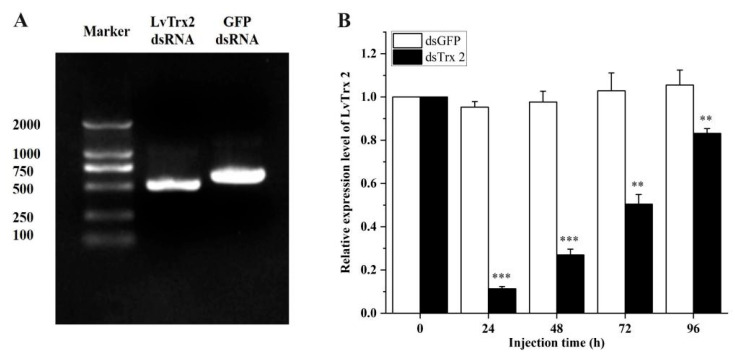
The quality of dsRNA synthesized in vitro (**A**) and the silencing efficiency of *LvTrx2* dsRNA injection (**B**). Statistical significance was calculated using SPSS 18.0 (** stands for *p* < 0.01, *** stands for *p* < 0.001).

The dsRNA of *LvTrx2* and *GFP* was synthesized using a T7 promoter-specific primer, which resulted in theoretical fragment sizes of 617 bp and 717 bp, respectively. The detection of synthetic target bands through agarose gel electrophoresis revealed the presence of single, high-quality synthesized bands without any additional bands (Figure 8A). The expression levels of *LvTrx2* in *dsLvTrx2*-injected shrimp decreased to less than 50% of the original level at 24 and 48 h post-injection, while the expression of *LvTrx2* remained constant in the shrimp injected with *dsGFP* (Figure 8B).

### 3.7. Expression Patterns in LvTrx2-Silenced Shrimp Following 4-NP Challenge

**Figure 9:**
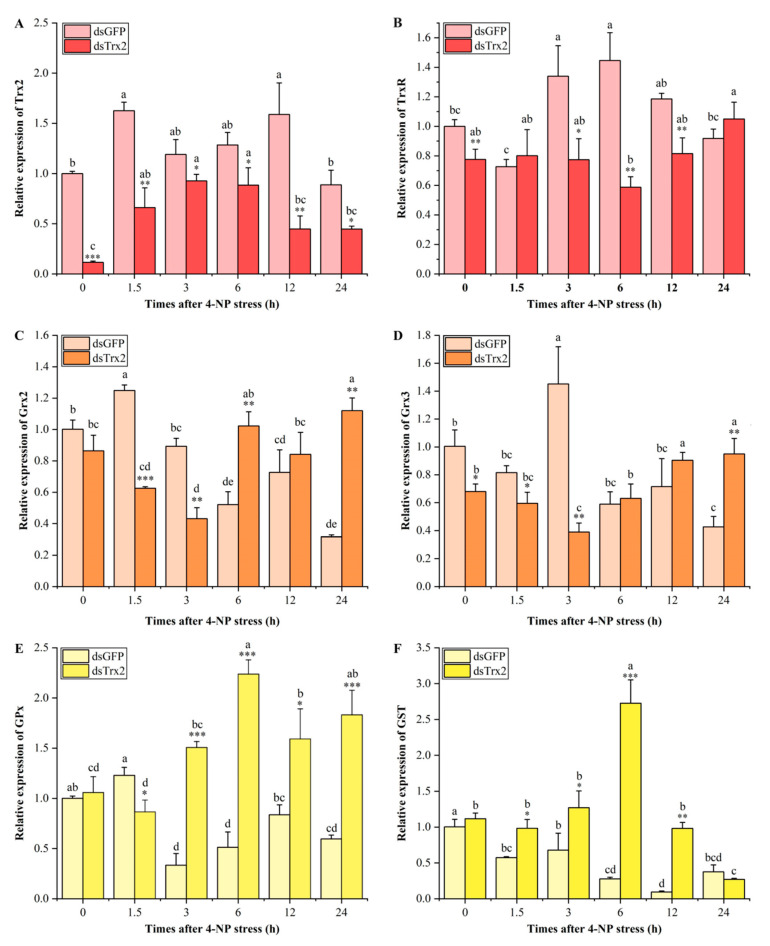
Expression levels of *LvTrx2* (**A**), *LvTrxR* (**B**), *Grx2* (**C**), *Grx3* (**D**), *GPx* (**E**), and *GST* (**F**) in the hepatopancreas of *LvTrx2*-interfered shrimp were detected under 4-NP stress *(n* = 9). Significant differences between the *dsTrx2* and *dsGFP* groups at the same exposure time are indicated by asterisks (* stands for *p* < 0.05, ** stands for *p* < 0.01, *** stands for *p* < 0.001). Data in the same group with different letters are significantly different (*p* < 0.05).

Twenty-four hours post-injection (designated as the 0 h of 4-NP exposure), the expression patterns of *LvTrx2*-related genes were detected under 4-NP stress. In the *dsGFP*-injected shrimp, the expression level of *LvTrx2* was significantly increased after 4-NP stress for 1.5 and 12 h (*p* < 0.05). In the *dsTrx2*-injected shrimp, the expression level of *LvTrx2* was significantly higher than that at 0 h after 1.5–6 h of 4-NP stress (*p* < 0.05). Compared to the *dsGFP* shrimp, the transcriptional level of *Trx2* in *dsTrx2*-interfered shrimp remained significantly lower at each time point after 4-NP exposure (*p* < 0.05), with the lowest expression observed at 0 h post-exposure (Figure 9A).

After 4-NP stress, the expression level of *TrxR* in the *dsGFP*-injected shrimp was significantly higher at 3 h and 6 h post-exposure than at 0 h (*p* < 0.05). In the *dsTrx2*-injected shrimp, there was no significant change in the expression level of *LvTrxR* during the whole process of 4-NP stress compared with that at 0 h stress (*p* > 0.05). Compared to the *dsGFP* shrimp, except for 1.5 h and 24 h, *TrxR* expression in the ds*Trx2*-injected group was lower than in the *dsGFP*-injected group (*p* < 0.05) (Figure 9B).

*Grx2* expression levels in the *dsGFP*-injected shrimp significantly increased at 1.5 h post-exposure, followed by a continuous decrease from 6 to 24 h after stress, reaching its lowest level at 24 h (*p* < 0.05). With the increase in interference time, the *Grx2* expression level first decreased and then increased. In the *dsTrx2*-injected shrimp, the expression level of *LvGrx2* showed a significant decrease after 3 h of 4-NP stress (*p* < 0.05). There was no significant difference in the expression level from 0 to 6–12 h (*p* > 0.05), followed by a significant increase after 24 h of stress (*p* < 0.05). Compared with the control group (*dsGFP*-injected), the *Grx2* expression level was significantly lower at 1.5 to 3 h while it was significantly higher than that at 6 and 24 h (*p* < 0.05) (Figure 9C).

In the *dsGFP*-injected shrimp, the expression level of *Grx3* was significantly increased after 3 h of 4-NP stress (*p* < 0.05) but significantly decreased at 24 h post-exposure (*p* < 0.05). In the ds*Trx2*-injected shrimp, the expression level of *Grx3* significantly decreased after 3 h of 4-NP stress (*p* < 0.05), followed by an upregulation from 12 to 24 h post-exposure (*p* < 0.05). Compared to the *dsGFP* group, the *Grx3* expression level in *dsTrx2*-injected shrimp was significantly inhibited at 0–3 h post-exposure (*p* < 0.05), while it exhibited a significant increase after 24 h of stress (*p* < 0.05) (Figure 9D).

In the *dsGFP*-injected shrimp, the expression level of *GPx* was significantly decreased at 3, 6, and 24 h under 4-NP stress (*p* < 0.05). In ds*Trx2*-injected shrimp, the expression level of *GPx* significantly increased (*p* < 0.05) from 6 to 24 h under stress, reaching its peak at 6 h. Compared to the *dsGFP* group, the expression level of *GPx* in *dsTrx2*-injected shrimp was significantly lower at the initial stage of stress (1.5 h), whereas it showed a significant increase during 3–24 h of stress (*p* < 0.05) (Figure 9E).

In the *dsGFP*-injected shrimp, the *GST* expression levels were lower from 0 to 24 h exposure (*p* < 0.05). In the *dsTrx2*-injected shrimp, a significant increase was observed after 6 h of stress, while a significant decrease was observed at 24 h (*p* < 0.05). Compared to the *dsGFP* shrimp, the *GST* expression level in *dsTrx2* shrimp was significantly higher at 1.5–12 h post-exposure (*p* < 0.05) (Figure 9F).

### 3.8. MDA Content in LvTrx2-Interfered Shrimp Under 4-NP Stress

**Figure 10:**
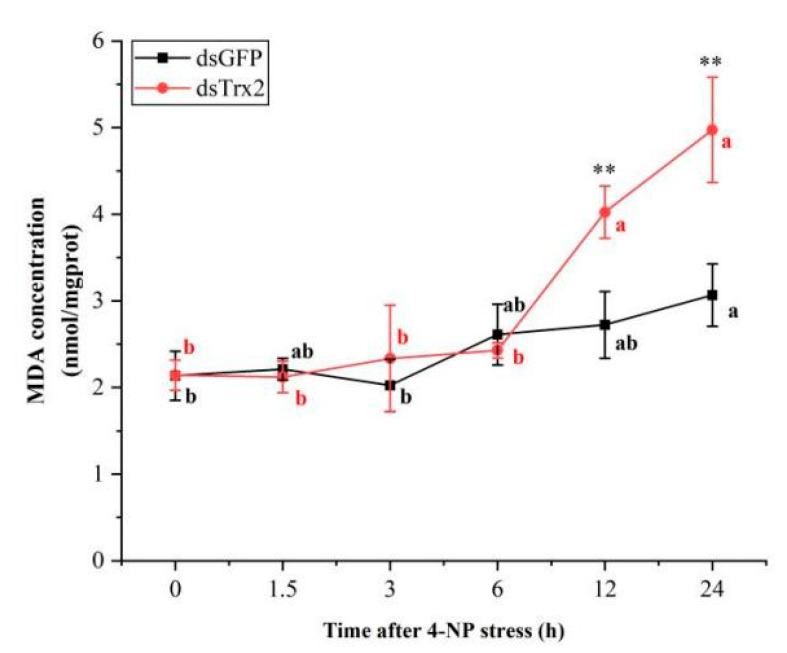
Time course of MDA contents in *LvTrx2*-interfered shrimp under 4-NP stress (n = 9). Significant differences between *dsTrx2-* and *dsGFP*-injected shrimp at the same exposure time are indicated with asterisks (** *p* < 0.01). Data in the same group with different letters are significantly different (*p* < 0.05).

In the *dsGFP* shrimp, the MDA content showed no significant change from 0 to 12 h of stress (*p* > 0.05), but it significantly increased at 24 h post-exposure (*p* < 0.05). In the ds*Trx2* shrimp, the level of MDA content was significantly increased at 12 and 24 h after 4-NP stress (*p* < 0.05). Compared to the *dsGFP* shrimp, the MDA content in *dsTrx2* shrimp was increased at 12 and 24 h post-exposure (*p* < 0.01) (Figure 10). 

## 4. Discussion

The Trx system is a crucial mechanism in living organisms, governing cellular redox reactions and playing a pivotal role in maintaining redox homeostasis within the cell [27]. *Trx* and *TrxR* are essential components of this system. In this study, we cloned the full-length sequences of *Trx2* and *TrxR* from *L. vannamei*, followed by subsequent molecular characterization analysis. The conserved domain and BLAST analyses revealed that the LvTrx2 protein belonged to the thioredoxin superfamily, exhibiting a highly stable active site sequence (W-C-G-P-C-K) that shares significant similarity with the Trx2 amino acid sequence of other species. Subcellular localization analysis shows that LvTrx2 possesses a mitochondrial targeting peptide, indicating its potential to enter mitochondria for functional purposes. In mammals, Trx has two distinct regulatory pathways: one is mediated by Trx1, predominantly localized in the cytoplasm and nucleus; the other is mediated by Trx2 found within mitochondria [11], consistent with previous results. The LvTrxR protein contains the conserved domain CVNVGC, which is unique to the TrxR family. Additionally, at the C-terminus of the LvTrxR protein, a GCUG sequence containing the rare amino acid Sec (U) exists as an active site incorporating Se. In the genetic code, the encoding of Sec is UGA, commonly used as a termination codon; if mRNA has a SElenoCysteine Insertion Sequence (SECIS), UGA becomes the encoding of Sec. Only a few enzymes contain this amino acid, such as TrxR, formate dehydrogenase, etc. Sec contains selenium alcohol groups that are more easily oxidized, and it has antioxidant activity in protein molecules [26].

The expression levels of *LvTrx2* and *LvTrxR* were detected in all of the tested tissues (gill, hepatopancreas, eyestalk, intestine, muscle, and hemocytes), indicating their involvement in fundamental metabolic processes. These findings are consistent with the results obtained from other species. In Kuruma shrimp (*Marsupenaeus japonicus*), *Trx* expression was observed in all six tissues [28]. The transcription levels of *Trx* and *Trx2* were detected in all six tissues examined in *S. paramamosain* [29]. The expression levels of both *Trx* and *TrxR*2 were found across all tissues of large yellow croaker (*Larimichthys crocea*) [30]. Although the presence of *Trx2* and *TrxR* is widespread in various tissues and organs, their distribution exhibits distinct tissue specificity. *M. japonicus* Trx was highly expressed in the gill and intestine [28]. In *S. paramamosain*, *Trx1* showed stronger expression in the gill and testis but displayed weaker expression in hemocytes [29], while *Trx2* expression levels were highest in the gill and hepatopancreas [31], which is consistent with the findings of *LvTrx2* in this study. In *L. crocea*, *TrxR*2 transcripts were highest in the gill and heart [30]. In the present study, *LvTrx2* and *LvTrxR* showed the highest levels of expression in both the hepatopancreas and gill. *Trx2* and *TrxR* exhibited different expression patterns in different tissues, which suggests that *LvTrx2* and *LvTrxR* possess a diverse range of biological functions in various tissues and organs. The hepatopancreas plays significant roles in immune response, digestion, metabolism, and detoxification processes in crustaceans. Gills are one of the most vital organs in crustaceans, serving primarily for gas and ion exchange with the external environment. Due to contact with the external environment, gills are susceptible to pathogen infections and environmental stress. Damage to gill tissues can result in the death of crustaceans. The high expression levels of *LvTrx2* and *LvTrxR* in the hepatopancreas and gill suggest their involvement in innate immunity and detoxification processes.

Trx plays an important role in defense against oxidative, hydrogen peroxide (H_2_O_2_) metabolism, immune response, anti-inflammatory, methionine sulfoxide reductases, transcription control, and signal transduction [11,32]. The first cloned rCcTrx1 protein possessed redox activity and protected against the oxidative damage of supercoiled DNA [33]. The overexpression of *MjTrx* in *M. japonicus* can increase the concentration of H_2_O_2_. *MjTrx* functioned in regulating redox homeostasis and shrimp antiviral immunity [28]. A different antioxidant response of *Trx* is elicited by the infectious hypodermal and hematopoietic necrosis (IHHNV) or white spot syndrome virus (WSSV) infectious process [34]. Different from *Trx*, *TrxR* is an essential player in antioxidant defenses, cellular redox systems, growth control, and selenium metabolism [35]. After selenium exposure in *O. mykiss* in vitro, *TrxR* was responsive to selenium exposure [18]. However, the functions of *Trx2* and *TrxR* in *L. vannamei* were not explicated or elucidated. This study aimed to investigate the role of *LvTrx2* and *LvTrxR* genes in mitigating environmental stress and pathogen infection by assessing their expression response under ammonia-N stress and LPS injection.

Ammonia-N, a prominent oxygen-consuming pollutant in aquatic environments, exerts detrimental effects on various aquatic organisms, including crustaceans, algae, and fish [36,37]. Ammonia-N induced metabolic and hematological dysfunction in Hong Kong oysters (*Crassostrea hongkongensis*) [38]. In *L. vannamei*, ammonia-N induced the injury of hepatopancreas and oxidative stress and increased the content of ROS [3]. In this study, the expression levels of *LvTrx2* and *LvTrxR* genes in different tissues exhibited different response patterns in response to different stresses or stimuli. Similarly to the observations in the big-belly seahorse (*H. abdominalis*), *HaTrx2* exhibited varying mRNA expression levels in response to immune challenges, which were dependent on both tissue type and time interval [20]. During ammonia-N stress, *LvTrx2* from the hepatopancreas exhibited a rapid response, leading to a significant upregulation within 3 h. In gill tissues, no significant changes or a certain degree of inhibition were observed during the early stage of stress. It was not until 48 h that the expression level of *LvTrx2* changed significantly. The molecular response of *LvTrx2* in the hepatopancreas was more sensitive compared to that in the gill following exposure to ammonia-N. These findings imply that *LvTrx2* plays a more significant role in the defense mechanisms of the hepatopancreas against ammonia-N stress compared to the gill. The hepatopancreas is a crucial organ involved in immune response, detoxification, and metabolism processes [39]. The upregulation of the *LvTrx2* expression level in the hepatopancreas can protect this organ. The gene expression level of *LvTrxR* was highly sensitive to ammonia-N stress. In the early stage of stress, the expression level of *LvTrxR* in the hepatopancreas and gills was significantly induced, followed by inhibition in the middle stage and subsequent re-induced upregulation in the later stage. The stimulation of macrophage lines and primary macrophage cultures from rainbow trout (*O. mykiss*) with pathogen-associated molecular patterns (PAMPs) results in the transcriptional induction of both *Trx* and *TrxR* during infection [18], which is consistent with the findings reported in this study. These findings suggest that *LvTrxR* may play an important role in the early and later stages of ammonia-N stress. Upon exposure to ammonia-N, *LvTrxR* expression in the hepatopancreas was approximately 14 times higher in the stress group compared to that in the control group, which indicates that the antioxidant protective effect of *LvTrxR* on hepatopancreas may be significant at this juncture. The accumulation of ammonia-N in the hepatopancreas contributes to severe damage, necessitating a rapid upregulation of *LvTrxR* to facilitate oxidative repair processes for proteins within this organ.

LPS is an integral component in the outer membrane of Gram-negative bacteria. LPS can reduce the metabolic ability of exogenous substances, induce inflammation, and reduce antioxidant capacity [40]. In our study, after LPS injection, the response mode of *LvTrx2* was different from that under ammonia-N stress. In hepatopancreas, the *LvTrx2* expression level was initially suppressed and then rapidly upregulated. In the gill tissue, the sustained upregulation of *LvTrx2* expression was observed in the mid-term. These findings indicate that *LvTrx2* is involved in the immune response against Gram-negative bacterial infection. The response of *LvTrx2* to the LPS challenge was more pronounced in the gill than in the hepatopancreas, with the highest relative expression level observed in the former. Similarly, the expression level of *LvTrxR* in the gill remained upregulated throughout the 48 h experiment, while in hepatopancreas, it significantly increased during the mid-term stage. When infected with LPS, *LvTrxR* plays an important role in the antioxidant defense process of gill, whereas it may contribute to antioxidant defense during the middle stage in hepatopancreas. Gill is an important organ for oxygen exchange in aquatic animals, which is crucial for their survival. The upregulation of *LvTrx2* and *TrxR* in the gill would protect this pivotal organ involved in respiration.

When *L. vannamei* encounter oxidative stress, they regulate the expression levels of antioxidant enzyme genes and activate the activity of antioxidant enzymes to respond to adverse environments [41]. Based on our previous research findings, 4-NP stress can induce oxidative stress in both the hepatopancreas and gills of *L. vannamei* [39,42]. Notably, *LvTrx2* exhibited significant upregulation in the hepatopancreas transcriptome under 4-NP stress. Therefore, *LvTrx2* was knocked down in vivo to verify the defense mechanism against 4-NP stress. The results indicated that, in comparison to the *GFP*-interfered group (control), the transcriptional levels of *LvTrx2* and *LvTrxR* were downregulated in *LvTrx2*-interfered shrimp. Both *Trx2* and *TrxR* belong to the Trx system and have an interactive relationship with each other. The expression of *Grx2* and *Grx3* decreased before exhibiting an increase at a later stage. In contrast, significant upregulation in the mRNA expression levels of *GPx* and *GST* was observed at multiple exposure times. The synergistic interaction between the thioredoxin system and the glutathione system suggests that *Trx* interference can lead to alterations in the expression of glutathione-related genes, thereby inducing *GST* expression via oxidative stress signaling pathways [43]. The MDA contents in *LvTrx2*-interfered shrimp were higher than those in the control group at a later stage (12 h and 24 h) after stress, which indicates that *LvTrx2* silencing increases the degree of oxidative damage. Trx interference can exacerbate lipid peroxidation and increase MDA levels [43]. These findings indicate a robust association between *LvTrx2* expression and other antioxidant-related genes, while *Grx2*, *Grx3*, *GPx*, and *GST* appear to compensate for the function of *LvTrx2*. These results suggest that *LvTrx2* plays an irreplaceable role in oxidative defense.

## 5. Conclusions

In this study, the full-length ORFs of *LvTrx2* and *LvTrxR* were successfully cloned from *L. vannamei* belonging to the thioredoxin system. *LvTrx2* and *LvTrxR* were expressed in all tested tissues, highly expressed in the hepatopancreas and gill. Through ammonia-N stress or LPS injection, the upregulation of the *LvTrx2* expression level in hepatopancreas and gill was induced to varying degrees, which indicates that *LvTrx2* is involved in the immune defense response process of stress resistance and antibacterial activity. Under ammonia-N stress and bacterial infection status, *LvTrxR* is involved in antioxidant defense processes in both hepatopancreas and gill. The *LvTrx2* expression silence experiment revealed that under 4-NP stress, the suppression of *LvTrx2* transcription caused an increase in the oxidative damage level in lipids and inhibited the expression levels of *Trx2* and *TrxR*. Both *Trx2* and *TrxR* belong to the Trx system and have an interactive relationship with each other. The expression levels of some antioxidant genes (e.g., *Grx2*, *Grx3*, *GPx*, and *GST*) were upregulated and may have a synergistic effect with *LvTrx2*, when *LvTrx2* was suppressed, which can play a compensatory role but cannot completely replace the function of *LvTrx2* and prove the importance of the defense function of *LvTrx2* under 4-NP stress.

## Figures and Tables

**Table 1:** The sequences of the primers used in the present study.

Primers	Gene ID	Sequences (5′–3′)
*Trx2*-F1	XM_027353791.2	ATTCTTCAACTCCTGTCGTG
*Trx2*-R1		TCTCCAATCAGCCTGCC
*TrxR*-F1	XM_027357384.1	GCTCCCGTGACCCTCAGTAA
*TrxR*-R1		CCAATGTGGTTTTGAATGCCTT
*Trx2*-3′F1		ATTCTTCAACTCCTGTCGTG
*Trx2*-5′R1		TGCCGACAAATGACTCAATCCG
*TrxR*-3′F1		AATGTGCCTACTACAGTGTTTACCCC
*TrxR*-5′R1		GCACATTGCCATAGTCGGTCAGC
3′RACE Outer Primer		TACCGTCGTTCCACTAGTGATTT
UPM long primer		CTAATACGACTCACTATAGGGCAAGCAGTGGTATCAACGCAGAGT
Short primer		CTAATACGACTCACTATAGGGC
*Trx2*-RT-F1		TGATAAGCAGCCTGATGGTG
*Trx2*-RT-R1		GCCTTGTTTCTGTTCCTCCA
*TrxR*-RT-F1		GTCGTCTGTTCCCCACATCTAT
*TrxR*-RT-R1		GCACATTGCCATAGTCGGTC
*Trx2*i-F (with T7)		GGATCCTAATACGACTCACTATAGGTCAGCGACGAATAGGGC
*Trx2*i-F		TCAGCGACGAATAGGGC
*Trx2*i-R (with T7)		GGATCCTAATACGACTCACTATAGGCAACATTTACGGAAGAGGGA
*Trx2*i-R		CAACATTTACGGAAGAGGGA
*GFP*i-F (with T7)		TAATACGACTCACTATAGGGAGAGTGCCCATCCTGGTCGAGCT
*GFP*i-F		GTGCCCATCCTGGTCGAGCT
*GFP*i-R (with T7)		TAATACGACTCACTATAGGGAGATGCACGCTGCCGTCCTCGAT
*GFP*i-R		TGCACGCTGCCGTCCTCGAT
*Trx1*-F	XM_027377405.2	TTAACGAGGCTGGAAACA
*Trx1*-R		AACGACATCGCTCATAGA
*Grx2*-F	MG757219	TGATAAGCAGCCTGATGGTG
*Grx2*-R		GCCTTGTTTCTGTTCCTCCA
*Grx 3*-F	XM_070127026.1	TTCAGCCGCACAACCATA
*Grx 3*-R		AGTCCTTGTCGCACTTCCTC
*GPx*-F	AY973252	AGGGACTTCCACCAGATG
*GPx*-R		CAACAACTCCCCTTCGGTA
*GST*-F	AY573381	AAGATAACGCAGAGCAAGG
*GST*-R		TCGTAGGTGACGGTAAAGA
*MGST3*-F	XM_027382186.2	TGTGCCGTTGGTGGTGC
*MGST3*-R		CAAGGAGCCCAGTAGCAAAAC
*β-Actin* F	AF300705	AACAGCGACTCCCGACAGA
*β-Actin* R		CCTTGGACCTAGTAATTTGCATG

Those underlined are the sequences of the T7 promoter.

## Data Availability

The partial data analyzed in this study are available from the corresponding author upon reasonable request.

## Supplementary Materials

The following supporting information can be downloaded at: https://www.mdpi.com/article/10.3390/ani15050629/s1.

## References

[1] FAO (2022). The State of World Fisheries and Aquaculture 2022.

[2] CAFS (2022). China Fishery Statistical Yearbook.

[3] Wang F.F., Huang L., Liao M.Q., Dong W.N., Liu C., Zhuang X.Q., Liu Y., Yin X.L., Liang Q.J., Wang W.N. (2021). Pva-miR-252 participates in ammonia nitrogen-induced oxidative stress by modulating autophagy in *Penaeus vannamei*. Ecotoxicol. Environ. Saf..

[4] Duarte-Restrepo E., Jaramillo-Colorado B.E., Duarte-Jaramillo L. (2020). Effects of chlorpyrifos on the crustacean *Litopenaeus vannamei*. PLoS ONE.

[5] Frías-Espericueta M.G., Abad-Rosales S., Nevárez-Velázquez A.C., Osuna-López I., Páez-Osuna F., Lozano-Olvera R., Voltolina D. (2008). Histological effects of a combination of heavy metals on Pacific white shrimp *Litopenaeus vannamei* juveniles. Aquat. Toxicol..

[6] Wang Z.L., Qu Y.X., Yan M.T., Li J.Y., Zou J.X., Fan L.F. (2019). Physiological Responses of Pacific White Shrimp *Litopenaeus vannamei* to Temperature Fluctuation in Low-Salinity Water. Front. Physiol..

[7] Kır M., Sunar M.C., Topuz M., Sarıipek M. (2023). Thermal acclimation capacity and standard metabolism of the Pacific white shrimp *Litopenaeus vannamei* (Boone, 1931) at different temperature and salinity combinations. J. Therm. Biol..

[8] Han S.Y., Wang B.J., Liu M., Wang M.Q., Jiang K.Y., Liu X.W., Wang L. (2018). Adaptation of the white shrimp *Litopenaeus vannamei* to gradual changes to a low-pH environment. Ecotoxicol. Environ. Saf..

[9] Powis G., Kirkpatrick D.L. (2007). Thioredoxin signaling as a target for cancer therapy. Curr. Opin. Pharmacol..

[10] Jia J.J., Xu G.T., Zhu D.S., Liu H.J., Zeng X.S., Li L. (2023). Advances in the Functions of Thioredoxin System in Central Nervous System Diseases. Antioxid. Redox Signal..

[11] Lu J., Holmgren A. (2014). The thioredoxin antioxidant system. Free Radic. Biol. Med..

[12] Vlamis-Gardikas A., Holmgren A. (2002). Thioredoxin and glutaredoxin isoforms. Meth. Enzymol..

[13] Hirota K., Nakamura H., Masutani H., Yodoi J. (2002). Thioredoxin superfamily and thioredoxin-inducing agents. Ann. N. Y. Acad. Sci..

[14] Zhang J. (2013). The Mitochondrial Thioredoxin Is Required for Liver Development in Zebrafish. Ph.D. Thesis.

[15] Kim D.H., Kim J.W., Jeong J.M., Park H.J., Park C.I. (2011). Molecular cloning and expression analysis of a thioredoxin from rock bream, *Oplegnathus fasciatus*, and biological activity of the recombinant protein. Fish Shellfish Immunol..

[16] Santhekadur P.K. (2020). Annual Reviews of Biochemistry. Curr. Sci..

[17] Hasan A.A., Kalinina E., Tatarskiy V., Shtil A. (2022). The Thioredoxin System of Mammalian Cells and Its Modulators. Biomedicines.

[18] Pacitti D., Wang T., Martin S.A., Sweetman J., Secombes C.J. (2014). Insights into the fish thioredoxin system: Expression profile of thioredoxin and thioredoxin reductase in rainbow trout (*Oncorhynchus mykiss*) during infection and in vitro stimulation. Dev. Comp. Immunol..

[19] Gu S.W., Zheng X.B., Gao X.M., Liu Y., Chen Y.E., Zhu J.Q. (2024). Cadmium-Induced Oxidative Damage and the Expression and Function of Mitochondrial Thioredoxin in *Phascolosoma esculenta*. Int. J. Mol. Sci..

[20] Nadarajapillai K., Sellaththurai S., Liyanage D.S., Yang H., Lee J. (2020). Molecular and functional explication of thioredoxin mitochondrial-like protein (Trx-2) from big-belly seahorse (*Hippocampus abdominalis*) and expression upon immune provocation. Fish Shellfish Immunol..

[21] Xu Z.H., Liu J., Wang E.M., Zhao C.Y., Hu X.L., Chu K.H., Wang L. (2021). Detoxification and recovery after cadmium exposure in the freshwater crab *Sinopotamon henanense*. Environ. Sci. Pollut. Res. Int..

[22] Lin Y.C., Chen J.C. (2001). Acute toxicity of ammonia on *Litopenaeus vannamei* Boone juveniles at different salinity levels. J. Exp. Mar. Biol. Ecol..

[23] Duan Y.F., Wang Y., Zhang J.S., Liu Q.S., Ding X. (2018). Morphologic, digestive enzymes and immunological responses of intestine from *Litopenaeus vannamei* after lipopolysaccharide injection. J. Invertebr. Pathol..

[24] Livak K., Schmittgen T. (2001). Analysis of Relative Gene Expression Data using Real-Time Quantitative PCR and the 2(-Delta Delta C(T)) Method. Methods.

[25] Xian J.A., Zhang X.X., Guo H., Wang D.M., Wang A.L. (2016). Cellular responses of the tiger shrimp *Penaeus monodon* haemocytes after lipopolysaccharide injection. Fish Shellfish Immunol..

[26] Alim I., Caulfield J.T., Chen Y., Swarup V., Geschwind D.H., Ivanova E., Seravalli J., Ai Y., Sansing L.H., Ste.Marie E.J. (2019). Selenium Drives a Transcriptional Adaptive Program to Block Ferroptosis and Treat Stroke. Cell.

[27] Xinastle-Castillo L.O., Landa A. (2022). Physiological and modulatory role of thioredoxins in the cellular function. Open Med..

[28] Guo N.N., Sun X.J., Xie Y.K., Yang G.W., Kang C.J. (2019). Cloning and functional characterization of thioredoxin gene from kuruma shrimp *Marsupenaeus japonicus*. Fish Shellfish Immunol..

[29] Hu J.H. (2014). Study on Thioredoxin System and Severalgenes Related to Antioxidant in Mud Crab, *Scylla paramam*. Master’s Thesis.

[30] Chen M.N. (2018). Identification and Function Analysis of Thioredoxin and Thioredoxin Reductase from *Larimichthys crocea*. Master’s Thesis.

[31] Hu J.H., Zhang F.Y., Jiang K.J., Fang Y.B., Wang J., Zhao M., Qiao Z.G., Ma L.B. (2014). Molecular characterization of thioredoxin-1 and thioredoxin reductase activity in mud crab *Scylla paramamosain*. Genet. Mol. Res..

[32] Pannala V.R., Dash R.K. (2015). Mechanistic characterization of the thioredoxin system in the removal of hydrogen peroxide. Biophys. J..

[33] Ruan Z.L., Liu G.Y., Guo Y.F., Zhou Y.H., Wang Q.Q., Chang Y.L., Wang B.L., Zheng J.M., Zhang L.M. (2017). First report of a thioredoxin homologue in jellyfish: Molecular cloning, expression and antioxidant activity of CcTrx1 from *Cyanea capillata*. PLoS ONE.

[34] Garcia-Orozco K.D., Sanchez-Paz A., Aispuro-Hernandez E., Gomez-Jimenez S., Lopez-Zavala A., Araujo-Bernal S., Muhlia-Almazan A. (2012). Gene expression and protein levels of thioredoxin in the gills from the whiteleg shrimp (*Litopenaeus vannamei*) infected with two different viruses: The WSSV or IHHNV. Fish Shellfish Immunol..

[35] Bjørklund G., Zou L., Peana M., Chasapis C.T., Hangan T., Lu J., Maes M. (2022). The Role of the Thioredoxin System in Brain Diseases. Antioxidants.

[36] Lin W., Wu J.Y., Luo H.M., Liu X.L., Cao B.B., Hu F., Liu F., Yang J.F., Yang P.H. (2023). Sub-chronic ammonia exposure induces hepatopancreatic damage, oxidative stress, and immune dysfunction in red swamp crayfish (*Procambarus clarkii*). Ecotoxicol. Environ. Saf..

[37] Liu S.Y., Luo L., Zuo F.Y., Huang X.L., Zhong L., Liu S., Geng Y., Ou Y.P., Chen D.F., Cai W.L. (2023). Ammonia nitrogen stress damages the intestinal mucosal barrier of yellow catfish (*Pelteobagrus fulvidraco*) and induces intestinal inflammation. Front. Physiol..

[38] Lu J., Yao T., Shi S.K., Ye L.T. (2022). Effects of acute ammonia nitrogen exposure on metabolic and immunological responses in the Hong Kong oyster *Crassostrea hongkongensis*. Ecotoxicol. Environ. Saf..

[39] Yu D.J., Zhai Y.F., He P.M., Jia R. (2022). Comprehensive Transcriptomic and Metabolomic Analysis of the *Litopenaeus vannamei* Hepatopancreas After WSSV Challenge. Front. Immunol..

[40] Wang Z.L., Wu Q.P., Liao G.W., Fan L.F. (2022). New insights into the regulation mechanism of *Litopenaeus vannamei* hepatopancreas after lipopolysaccharide challenge using transcriptome analyses. Fish Shellfish Immunol..

[41] Guo H., Xian J.A., Li B., Ye C.X., Wang A.L., Miao Y.T., Liao S.A. (2013). Gene expression of apoptosis-related genes, stress protein and antioxidant enzymes in hemocytes of white shrimp *Litopenaeus vannamei* under nitrite stress. Comp. Biochem. Physiol. C Toxicol. Pharmacol..

[42] Su X.B., Li T., Zhu X.W., Zheng P.H., Pan H.K., Guo H. (2024). Exploring the impact of nonylphenol exposure on *Litopenaeus vannamei* at the histological and molecular levels. Ecotoxicol. Environ. Saf..

[43] Holmgren A., Lu J. (2010). Thioredoxin and thioredoxin reductase: Current research with special reference to human disease. Biochem. Biophys. Res. Commun..

